# CyFiT telehealth: protocol for a randomised controlled trial of an online outpatient physiotherapy service for children with cystic fibrosis

**DOI:** 10.1186/s12890-019-0784-z

**Published:** 2019-01-24

**Authors:** Ray Lei Lang, Christine Wilson, Kellie Stockton, Trevor Russell, Leanne Marie Johnston

**Affiliations:** 1 The University of Queensland School of Health and Rehabilitation Sciences, Building 84A, The University of Queensland, St Lucia, Queensland 4072 Australia; 2 Children’s Health Queensland Hospital and Health Services, 501 Stanley Street, South Brisbane, Queensland 4101 Australia

**Keywords:** Cystic fibrosis, Telehealth, Telemonitoring, Children, Health economics, Physical activity

## Abstract

**Background:**

Telehealth and telemonitoring is an emerging area of study in people with cystic fibrosis (CF), with the potential of increasing access to care, and minimising infection control risks to patients without compromising their health outcomes. To date, limited evidence is available to support the use of telehealth in paediatric population with CF in a clinical setting. This study aims to investigate the utility of a multimodal telehealth-based outpatient physiotherapy service and assess its effect on quality of life, functional exercise capacity, hospital admission and intravenous antibiotic requirements, lung function, processes of care, participation in activities of daily living, and health economics associated with operating an innovative service.

**Method:**

This single centre, prospective, parallel, randomised, controlled, non-inferiority trial aims to recruit 110 children with CF between the ages 8 to 18 years of age. Participants will be randomised to the Usual Outpatient Physiotherapy Service group (Usual OPS) or the telehealth intervention group (CyFiT OPS). Quality of life, participation in activity of daily living, functional exercise capacity and patient perception of care will be examined every six months using the Cystic Fibrosis Questionnaire-Revised (CFQ-R), Children’s Assessment of Participation and Enjoyment (CAPE), Preferences for Activities of Children (PAC) questionnaire, Modified Shuttle Test-25 (MST25), and Measure of Process of Care (MPOC-20) questionnaire. Physiological measurements collected during routine clinical visits such as spirometry, body weight and height, information will be retrospectively retrieved via a chart review at the end of the study.

**Discussion:**

We anticipate that this multi-modal telehealth service will deliver a comparable service to traditional face-to-face models. An alternative to existing outpatient physiotherapy services may potentially increase patient options for access to care and patient-orientated outcomes such as quality of life. If deemed appropriate, the new model of care can be integrated into clinical practice immediately.

**Trial registration:**

This trial is registered with the Australian and New Zealand Clinical Trial Registry (ACTRN12617001035314) last updated 17th July 2018.

**Electronic supplementary material:**

The online version of this article (10.1186/s12890-019-0784-z) contains supplementary material, which is available to authorized users.

## Background

Cystic fibrosis (CF) is an inherited disorder of exocrine function that affects the lungs, digestion, and multiple other body systems. Ongoing physiotherapy in the form of airway clearance techniques and exercise therapy is integral to the management of CF [1]. Adherence to long-term physiotherapy regimens, however, can be difficult at home [2–4]. Poor adherence can lead to more frequent visits to hospital, which potentially increases exposure to harmful infections at the hospital, as well as time away from school and work. At our clinic, physiotherapists utilise outpatient physiotherapy services (OPS) in addition to regular physiotherapy reviews during multidisciplinary (MDT) clinics to encourage prescribed physical activity and effective airway clearance techniques [1]. Traditionally, OPS are delivered via face-to-face consultations in a hospital setting. An alternative mode of delivering OPS is therefore needed to enable better access to healthcare and minimise risk of cross infection. This study aims to investigate the efficacy of an innovative telehealth-based outpatient physiotherapy service for children with CF.

Telehealth in the form of real-time videoconferencing enables patients to access health care from virtually anywhere. The risk of cross infection may be reduced as patients are not required to visit hospital as frequently. Other forms of telehealth may improve patient experiences and better clinical outcomes in ways that were previously not possible. For example, virtual group exercise may offer much needed peer-driven social/psychological support between patients as children are currently unable to physically interact with other children with CF due to infection precautions [1]. In addition, smartphone and wearable technologies may assist physiotherapists in clinical decision making as they are able to access a large amount of health-related data remotely [5].

To date, the role of telehealth in the physiotherapy management of CF is unclear, although various centres internationally are experimenting with different types of telehealth interventions. Telehealth in the form of telemonitoring via spirometry have previously been investigated [6–8]. More recent publications have suggested the use of telehealth to increase access to health care (Jamie [9]), as well as encouraging physical activity [10], and mobile-based applications ([11]; J. [12]) in adults with CF. Limited research is available for children with CF.

We aim to investigate the efficacy of various modes of telehealth at our clinic. In this trial, we have developed a multi-modal telehealth-based OPS specific for children with CF. This trial seeks to answer the following question: Can CyFiT OPS offer a non-inferior health outcome and quality of life compared to Usual OPS for children with CF? If proven to be effective, the new model of healthcare delivery will be integrated into our existing services as an alternative option for families particularly those living far away from the hospital CF Centre.

## Methods/design

### Study aim

The aim of this study is to investigate the efficacy of a telehealth outpatient physiotherapy service (CyFiT OPS) on patient quality of life, clinical efficacy and cost-effectiveness of healthcare delivery when compared to Usual OPS.

### Study design

This is a single centre, prospective parallel randomised controlled non-inferiority trial with a 1:1 allocation across two treatment arms: Usual OPS and CyFiT OPS. Children receiving Usual OPS will serve as the control group for the trial.

### Study setting

All trial activities will be conducted through the Children’s Health Queensland Hospital and Health Service (CHQ-HHS) CF service in Queensland Australia. The CF service operates from Lady Cilento Children’s Hospital (LCCH), South Brisbane, Queensland, Australia and outreach service locations in Queensland.

### Participants

#### Inclusion criteria

Participants will be children (i) with a confirmed medical diagnosis of CF, (ii) aged between eight to eighteen years (inclusive), (iii) who have access to the internet in their local area (e.g. at home, a local health centre, or the home of a family-selected relative or friend), (iv) through a device which enables videoconferencing (e.g. personal computer, tablet, or phones).

#### Exclusion criteria

Children will be excluded from this study if they present with (i) an acute or chronic medical co-morbidity that would prevent or significantly limit participation in the study, or (ii) behavioural or intellectual difficulties that would prevent full participation in face-to-face physiotherapy assessment, or physiotherapy intervention via telehealth, or (iii) they are involved in another study that precludes enrolment in any other study.

### Recruitment process

Participants of this study will be recruited from the CHQ-HHS CF service, Queensland, Australia. This service is provided via inpatient and outpatient services at (i) the CHQ-HHS LCCH, (ii) regional Queensland Health hospitals.

Children will be identified by the study coordinator who is a senior physiotherapist will review each patient attending the CF clinic, OPS, or hospital ward daily. The inclusion and exclusion criteria will be used to determine the eligibility for each child. All eligible children will be approached by the treating physiotherapist. If families express interest in the trial, a member of the research team will provide written information and seek consent/assent as appropriate. If the parents agree for their child to proceed with the study, informed written consent will be gained from the parent and assent from the child.

Once informed written consent is gained, baseline assessment will be collected by the blinded research assessor, which includes seven elements, the:, (i) Cystic Fibrosis Questionnaire-Revised (CFQ-R) (A. [13]), (ii) Modified Shuttle Test (MST25) [14], (iii) Measure of Processes of Care (MPOC) Questionnaire (S. M. [15]), (iv) Children’s Assessment of Participation and Enjoyment (CAPE), (v) Preferences for Activities of Children (PAC) (G. A. [16]), (vi) lung function (i.e. FEV_1_, FVC, FEV_1_/FVC, FEF_25–75%_)[17], and (vii) anthropometric data: height (cm), weight (kg) and age (y).

Once baseline assessment has been scheduled, a physiotherapist will randomise the child to one of the two treatment arms by opening a sealed allocation envelope in front of the child. The physiotherapist will then discuss with the families the requirements relevant to their allocation (e.g. how to access the telehealth system).

### Randomisation

Children will be randomised via concealed random allocation. A computerised random number sequence will be generated that corresponds to the two study arms (1 = Usual OPS, 2 = CyFiT OPS) in balanced-blocks of eight participants (i.e. 4 Usual OPS, 4 CyFiT OPS). The result of each randomisation will be concealed inside a sequentially numbered, opaque envelope. Intervention will be provided to each child in one of two treatment arms: Usual OPS or CyFiT OPS. The allocation of each child will be recorded on an electronic, password protected spreadsheet that is unknown to the trial assessor, and then contents of the envelope will be discarded.

### Intervention

Usual physiotherapy management for each child in the study comprises 3 components:

1. ***Multi-disciplinary (MDT) Review & Individual Exacerbation Plan:***
  As recommended by Australian and New Zealand clinical practice guidelines [1], all participants in both groups will receive one face-to-face physiotherapy session in a multidisciplinary CF clinic approximately every three months. Each child with CF is advised on an individualised exacerbation plan where minor exacerbations can be managed at home with parental supervision.
2. ***Outpatient physiotherapy services (OPS)*** **:**
  Apart from these routine check-ups, OPS may be clinically indicated on an individual patient basis. Timing and frequency of OPS will be determined by the child’s treating physiotherapist according to clinical indicators which will be identical for children in both treatment arms. If OPS is required, it will be delivered in a mode according to the group that the child has been randomised to - Usual OPS or CyFiT OPS. Physiotherapy in both arms will be delivered by the same physiotherapy team. Only the mode of delivering OPS will differ according to group allocation (face-to-face or telehealth).
3. **Escalated Care with Hospital Admission:**
  If a child in either group presents with any clinical indicator for admission to the hospital, then the child will receive face-to-face physiotherapy by hospital physiotherapy staff during these periods of inpatient admission as per routine clinical practice.

#### Usual outpatient physiotherapy services (usual OPS)

Participants allocated to the Usual OPS group may receive OPS as a combination of face-to-face, telephone, and/or telehealth follow up as determined at the time by the treating physiotherapist. Each review may involve any or a combination of the child’s home exercise program, airway clearance techniques, use and maintenance of therapy equipment, and support for adherence. If telehealth is included as an intervention mode, this will be delivered through the standard Queensland Health Telehealth Network (QH Telehealth). The QH telehealth system enables physiotherapists to connect via real-time videoconferencing. Physiotherapists may choose to utilise QH Telehealth to replace some face-to-face reviews to better suit patient schedules or to provide video feedback (e.g. seeing child demonstrating airway clearance techniques) that is otherwise not possible through a telephone review.

#### CyFiT outpatient physiotherapy (CyFiT OPS)

Participants allocated to the CyFiT OPS group will receive OPS via telehealth as the primary mode of delivery. In this group, a different telehealth system called eHAB® will be used. The eHAB® system was developed at The University of Queensland for specialised telerehabilitation. When using eHAB® with an individual child, clinicians can access additional digital functions not available via existing QH Telehealth System, such as real-time image/video sharing, whiteboard for drawing diagrams, measurement tools for looking at various joint ranges of motion across the body and session recording that will facilitate an enhanced telehealth experience compared to videoconferencing alone. In addition, eHAB® enables clinicians to work with groups of children, for example to host real-time multipoint video conferences to deliver virtual group-based sessions.

In addition to having access to the eHAB® telehealth system for CyFit OPS, children allocated to the CyFiT group will be asked to wear a consumer-based wrist-worn activity tracker, Garmin Vivosmart3® (Garmin Ltd., Lenexa, KS, USA), during the 12-month intervention period. Children can utilise all functions available on the activity tracker. Health-related data (such as heart rate variability, sleep duration, steps, distance travelled, energy expenditure) will also be monitored by the treating team to inform physiotherapy practices in exercise prescription and in telehealth sessions (with access to near-real-time physiological information).

##### Integration of CyFiT into current model of care

Physiotherapists will have access to health-related data collected from wrist-worn activity trackers. This information can be used to support clinical decision making by physiotherapists, to initiate OPS check-up (in the form of telehealth) and feedback to the multidisciplinary team. From the patient’s perspective, health-related data displayed on their activity trackers may assist them in self-managing their therapy.

#### Clinician support system

This trial will utilise a web-based clinician support system developed by The University of Queensland. This system will enable physiotherapists to monitor health-related data collected from activity wristbands, organise online group-based sessions and engage with families remotely for children in CyFiT OPS. Children with sudden changes to health-related data will be automatically highlighted to clinicians via the clinician support system. Changes in health-related data may then be used by physiotherapists to assist in clinical decision-making and initiate additional follow-up.

### Study outcomes

#### Study duration

Each child will be involved in the trial for 18 months, including a 12-month intervention period where the child is randomised to either Usual OPS or CyFiT OPS, followed by a 6 month follow up period where all participants will revert to Usual OPS and use of QH Telehealth services (as indicated). In CyFiT OPS, the intervention period can be further divided into phase one (0-6 months) and phase two (6-12 months) (see below). All outcome measures will be assessed (i) at baseline, (ii) after 6 months, (iii) after 12 months - end of intervention, and (iv) at 18 months – 6 months after the end of intervention, for both groups.

##### Phase one

Phase one is an opportunity for children and clinical physiotherapists to familiarise themselves with technology (i.e. activity wristbands and eHAB®). During this period, children will only be participating in one-to-one OPS via telehealth in the intervention group as clinically indicated. Treating physiotherapists will have access to health-related data via supplier web portal. Health-related data collected during phase one will enable the research team to derive statistical models to monitor children’s health and better assist clinical decision making.

##### Phase two

Phase two occurs in the last 6 months of the 12-months intervention. In addition to one-on-one OPS via telehealth, physiotherapists can refer children in the CyFiT OPS group to an online group-based exercise class. Group sessions will be delivered using the eHAB® system. In this phase, health-related data and risk score will be visualised using the clinician support system, and this information will be used by physiotherapists to assist in clinical decision making, such as initiating OPS check-ups.

#### Primary outcome measure

##### Cystic fibrosis questionnaire – Revised (CFQ-R)

The CFQ-R is a valid and reliable quality of life measure for children with CF from 6 years of age (A. L. [18]). The score is positively correlated with pulmonary function in adolescents with CF [19]. It includes nine quality of life domains: physical (8 items), role limitation, vitality (5 items), emotional (5 items), social (6 items), body image (3 items), eating disturbances (3 items), treatment burden (3 items), and health perceptions (3 items); as well as three symptom-based scales: weight (1 item), respiratory (6 items), digestion (3 items). All items are scored on a 4-point Likert Scale (1 = Very true/Always to 4 = Not at all true/Never). Scores for each domain will be analysed individually.

#### Secondary outcome measures

##### Modified shuttle test

The modified shuttle test (MST25) is an externally paced maximal exercise capacity test that has been validated with peak aerobic capacity in children with CF [14]. Our clinic regularly employs the MST as an outcome measure secondary to its high reliability [20] and responsiveness to physiotherapy interventions [21]. Heart rate (bpm), oxygen saturation (SpO_2_ in %), rate of perceived exertion (modified Borg dyspnoea scale) [22], and 15-count breathlessness score [23] will be collect at four timepoints: pre-MST25, immediately post-MST25, 1 min recovery, and 2 min recovery. Distance (metres), level completed and self-reported limiting factor (e.g. breathlessness, leg fatigue) will be collected.

##### Medical record information

A chart review of medical records will be performed to extract medical information such as: hospital, outpatient appointments (face-to-face and/or telehealth), number and type of staff patient seen, and intravenous antibiotic days as well as clinical measurements collected routinely in three monthly MDT reviews: body height, weight, age, spirometry, medication, and relevant social factors (e.g. distance travelled to attend clinic / hospital).

##### Cough and activity questionnaire

Self-reported cough and activity questionnaires will be completed weekly. Parameters of cough frequency, moistness, and colour of sputum will be collected (Additional file 1: Appendix 1).

##### Participation

The CAPE and PAC are questionnaires that measure participation of children in a range of activities outside of school. The CAPE and PAC collects information about the child’s frequency, diversity, and enjoyment of an activity in formal (organised sport, other skill-based activities as well as clubs, groups and organisations) and informal activities (recreational, active-physical, social, skill-based and self-improvement).

##### Quality of care

The Measure of Processes of Care (MPOC) assessment is a validated and reliable questionnaire tool useful in understanding parents’ perceptions of the care they and their children receive (S. M. [15]). The MPOC addresses five aspects of care: Enabling and Partnership, Providing General Information, Providing Specific Information about the Child, Coordinating and Comprehensive Care for the Child and Family, and Respectful and Supportive Care. Data from all five domains will be collected and analysed.

### Cost effectiveness

Economic analysis will be performed from both the healthcare and patient perspective. Costs associated with health care services will be retrospectively retrieved post intervention (e.g. cost of outpatient visits, inpatient admissions, staff time and equipment). Healthcare costs from the patient’s perspective will be calculated using data collected from a chart audit (e.g. number of hospital visits, and services utilised), additional information will be estimated (e.g. costs associated with commuting, time taken off work, and number of family members who accompany the child to clinic). Additional information will be estimated by investigators using published data (e.g. cost of fuel, duration of travel). Incremental cost-effectiveness ratio between the two groups will be calculated using the CFQ-R. Cost per reduction in hospital days will also be analysed if a difference between the two groups is observed.

### Blinding

The nature of this trial prevents the blinding of both the treating clinicians and patients. Both clinicians and patients will be exposed to the allocation via a) what modality they receive their physiotherapy service, and b) health-related data from activity trackers. Outcome measures will be collected by a blinded assessor. Randomisation will only occur after initial baseline assessment. Patients will always be approached by an unblinded clinical team prior to reassessment to mitigate risk of unblinding of the assessor (e.g. removing wristbands for the assessment).

### Sample size

The minimum clinically important difference (MCID) for the CFQ-R has been previously calculated for people with CF who are medically stable (using distribution/Smallest Error Measurement method) as 6.1 (A. L. [24]). Standard deviation on CFQ-R improvement (treatment burden) has been previously reported as 8.6 [25]. A power of 0.9 and an alpha value of 0.025 has been chosen for this trial. Using this information, sample size for each group has been calculated to be 42 participants per group [26]. To allow for patient dropout, a total of 55 participants per group will be recruited.

### Data analysis

All statistical analyses will be performed using SPSS 24 (IBM Corp., Armonk, NY, USA). Data will be analysed on an intention-to-treat basis. Generalised linear models will be used to determine any differences between repeated outcome measures across Usual OPS and CyFiT OPS. Missing data will be handled using predictive mean matching imputations using five observed cases for each missing value and a total of five imputed data sets will be pooled as the final result [27]. Statistical significance will be set at *p* < 0.05.

### Withdrawal

Participants are considered withdrawn when consent is revoked. Withdrawn participants will not undertake any further assessment or intervention. Data collected up until point of withdrawal will form part of the study result. Participants will not be withdrawn if not adhering to protocol. Withdrawal from the trial will have no impact on the care offered to families.

### Data storage

All patient outcome measures will be collected in paper format and stored in a locked cabinet at Lady Cilento Children’s Hospital with restricted access. Data from wearable activity trackers are stored by Garmin (Garmin Ltd., Lenexa, KS, USA); participants will give the investigators permission to download that information to the University of Queensland servers for the use of Clinician Support System. Trial data will be stored electronically on a password protected Queensland Health Server for a period of 15 years in accordance with NHMRC guidelines. Data will be checked and cleaned prior to being locked for analysis.

### Data monitoring

The research team will meet every six months to ensure that recruitment is on schedule and performed ethically, as well as to assess any adverse events associated with the intervention. Any adverse events will be immediately notified to the overseeing ethics committee (Children’s Health Queensland) and the site-relevant governance committee, and the TSC. In occurrences where safety concern is identified by the research team, a suspension of the trial can be issued by the research team and a recommendation to the overseeing ethics and site governance committee to modify or stop the trial will be made.

### Treatment Fidelity

All treating physiotherapists involved in the trial will undergo a training session on the use of the eHAB®, Garmin Connect (Garmin Ltd., Lenexa, KS, USA), and Clinician Support System led by the research team. Handouts and manuals will be developed by the research team and easily accessible to the clinical team. The research team will be in clinic on a regular basis to maintain adherence to protocol.

## Discussion

**Fig. 1:**
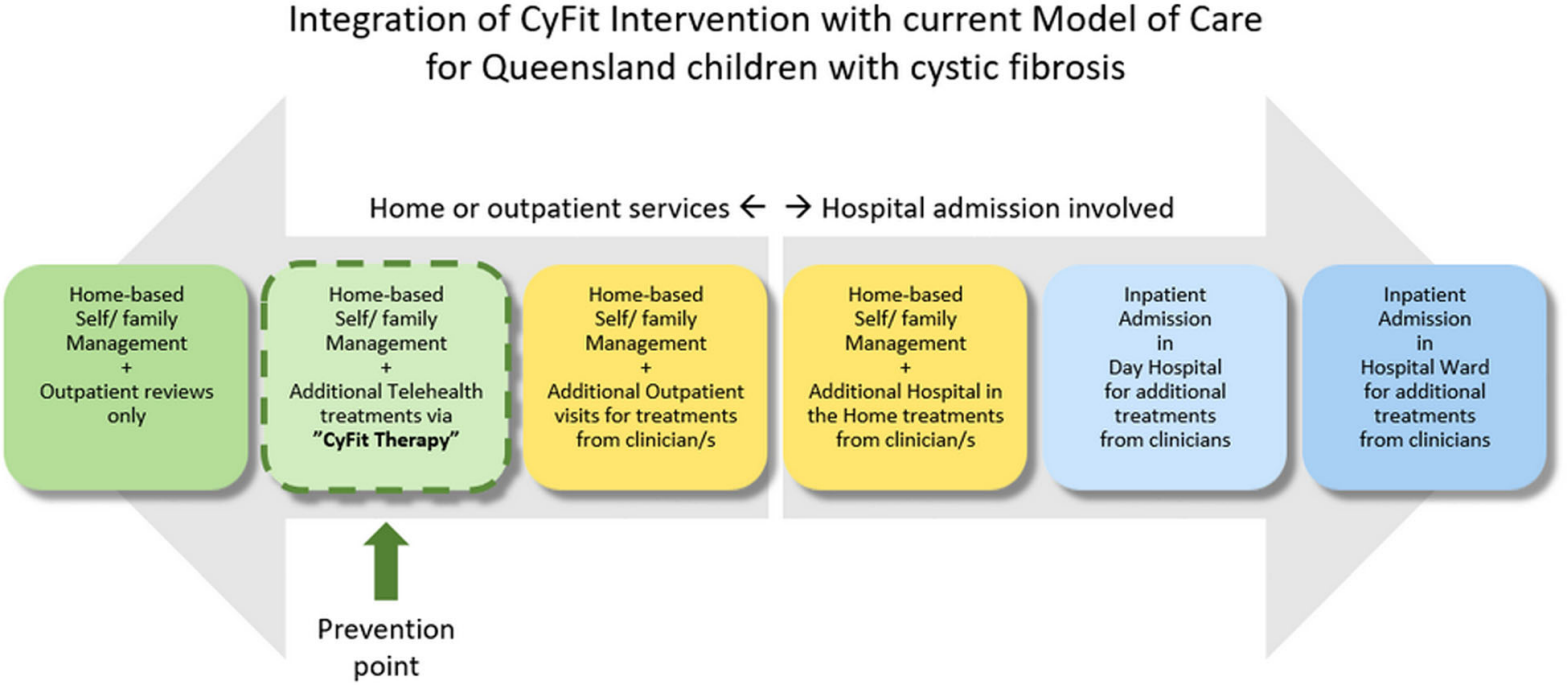
Integration of CyFiT Intervention with current Model of Care

To the best of the authors’ knowledge, this study is the first in the world to assess the efficacy of a telehealth-based outpatient physiotherapy clinic for children with CF. Emerging literature supports the efficacy of online telehealth clinics in offering increased access to care for adults with cystic fibrosis ([10]; Jamie [9]) and remote monitoring for deteriorating health [8]. In previous studies, researchers have examined each innovation independently as an alternative to current practices. In this study, researchers will utilise these innovations as an adjunct to existing services (Fig. 1). The new model of care will enable clinicians to better facilitate ongoing intervention programs in the future.

Remote monitoring of physical activity and health-related data is an essential part of the proposed model. Previously, activity diaries have been used to monitor physical activity, however these are self-reported and often inaccurate [28]. The use of wrist-worn activity trackers should alleviate such inaccuracies. Activity trackers continuously collect health-related data passively in near-real-time, which will provide more data. Consumer-based activity trackers have been chosen over clinical/research counterparts to increase acceptance from children in the trial.

The large amount of data that will be available for clinicians raises new challenges. The sheer volume of data from activity trackers have previously made near-real-time analysis impractical and unusable in a clinical setting. To minimise this, a clinician support system will be developed for this study to facilitate the near-real-time statistical modelling and display of information in a cost-effective manner.

Study bias has been minimised such that the research team and assessor are blinded from participant allocation and are not directly involved in the clinical management of participants.

Telehealth-based physiotherapy consultations enable children and physiotherapists to access more diverse and engaging options of therapeutic intervention. Telehealth enables online group sessions where multiple children can participate in a session together without the risk of cross infection. Social-based activities and gamification elements offered by consumer-based activity trackers may reinforce physical activity habits in children. Identifying practical means of continually engaging and facilitating children in participating in airway clearance and activities of daily living will yield long-term benefits for children with CF.

In conclusion CyFiT OPS is formulated around a patient-centred model. Establishing the clinical utility of CyFiT OPS means that families can receive the same quality of physiotherapy service closer to home, with a reduced risk of cross infection. This study will also supplement existing literature by providing high quality evidence regarding the use of telehealth in managing children with CF.

## Additional file

Additional file 1: **Figure 1.** Illustrates the current model of physiotherapy care for children with cystic fibrosis at our clinic. Prevention point is where the study intervention will be integrated into existing practices. (DOCX 133 kb)

## Abbreviations

CAPE: Children’s Assessment of Participation and Enjoyment
CF: Cystic Fibrosis
CFQ-R: Cystic Fibrosis Questionnaire – Revised
CHQ-HHS: Children’s Health Queensland Hospital and Health Services
FEF_25–75_: Forced Expiratory Flow at 25–75%
FEV1: Forced Vital Volume in One Second
FEV1/FVC: Forced Vital Volume in One Second divided by Forced Vital Capacity
LCCH: Lady Cilento Children’s Hospital
MCID: Minimal Clinical Important Difference
MDT: Multi-disciplinary Team
MPOC: Measure of Processes of Care
MST25: Modified Shuttle Test with 25 levels
NHMRC: National Health and Medical Research Council (Australia)
OPS: Outpatient Physiotherapy Service
PAC: Preferences of Activity of Children Questionnaire
QH: Queensland Health

## References

[1] Button BM, Wilson C, Dentice R, Cox NS, Middleton A, Tannenbaum E (2016). Physiotherapy for cystic fibrosis in Australia and New Zealand: a clinical practice guideline. Respirology.

[2] Arias Llorente RP, Bousono Garcia C, Diaz Martin JJ (2008). Treatment compliance in children and adults with cystic fibrosis. J Cyst Fibros.

[3] Passero MA, Remor B, Salomon J (1981). Patient-reported compliance with cystic fibrosis therapy. Clin Pediatr (Phila).

[4] Sawicki GS, Ren CL, Konstan MW, Millar SJ, Pasta DJ, Quittner AL (2013). Treatment complexity in cystic fibrosis: trends over time and associations with site-specific outcomes. J Cyst Fibros.

[5] Tagliente I, Solvoll T, Trieste L, De Cecco CN, Murgia F, Bella S (2016). Which indicators for measuring the daily physical activity? An overview on the challenges and technology limits for telehealth applications. Technol Health Care.

[6] Aarti, S., Niko, K., Lauren, K., & Z., N. S. (2018). Impact of home spirometry on medication adherence among adolescents with cystic fibrosis. Pediatr Pulmonol*,* 53(4), 431–436. doi:doi:10.1002/ppul.23950 10.1002/ppul.23950 29457700

[7] Choyce J, Shaw KL, Sitch AJ, Mistry H, Whitehouse JL, Nash EF (2017). A prospective pilot study of home monitoring in adults with cystic fibrosis (HOME-CF): protocol for a randomised controlled trial. BMC Pulm Med.

[8] Cox NS, Alison JA, Rasekaba T, Holland AE (2012). Telehealth in cystic fibrosis: a systematic review. J Telemed Telecare.

[9] Wood J, Mulrennan S, Hill K, Cecins N, Morey S, Jenkins S (2016). Telehealth clinics increase access to care for adults with cystic fibrosis living in rural and remote Western Australia. J Telemed Telecare.

[10] Cox NS, Alison JA, Button BM, Wilson JW, Holland AE (2015). Feasibility and acceptability of an internet-based program to promote physical activity in adults with cystic fibrosis. Respir Care.

[11] Calvo-Lerma J, Martinez-Jimenez CP, Lazaro-Ramos JP, Andres A, Crespo-Escobar P, Stav E (2017). Innovative approach for self-management and social welfare of children with cystic fibrosis in Europe: development, validation and implementation of an mHealth tool (MyCyFAPP). BMJ Open.

[12] Wood J, Jenkins S, Putrino D, Mulrennan S, Morey S, Cecins N, Hill K. High usability of a smartphone application for reporting symptoms in adults with cystic fibrosis. J Telemed Telecare. 2017:1357633x17723366. 10.1177/1357633x17723366. 10.1177/1357633X17723366 28799841

[13] Quittner A, Suthoff E, Rendas-Baum R, Bayliss MS, Sermet-Gaudelus I, Castiglione B, Vera-Llonch M (2015). Effect of ivacaftor treatment in patients with cystic fibrosis and the G551D-CFTR mutation: patient-reported outcomes in the STRIVE randomized, controlled trial. Health Qual Life Outcomes.

[14] Selvadurai HC, Cooper PJ, Meyers N, Blimkie CJ, Smith L, Mellis CM, Van Asperen PP (2003). Validation of shuttle tests in children with cystic fibrosis. Pediatr Pulmonol.

[15] King SM, Rosenbaum PL, King GA (1996). Parents' perceptions of caregiving: development and validation of a measure of processes. Dev Med Child Neurol.

[16] King GA, Law M, King S, Hurley P, Hanna S, Kertoy M, Rosenbaum P (2007). Measuring children's participation in recreation and leisure activities: construct validation of the CAPE and PAC. Child Care Health Dev.

[17] Solem CT, Vera-Llonch M, Liu S, Botteman M, Castiglione B (2016). Impact of pulmonary exacerbations and lung function on generic health-related quality of life in patients with cystic fibrosis. Health Qual Life Outcomes.

[18] Quittner AL, Buu A, Messer MA, Modi AC, Watrous M (2005). Development and validation of the cystic fibrosis questionnaire in the United States: a health-related quality-of-life measure for cystic fibrosis. Chest.

[19] Thomas C, Mitchell P, O’Rourke P, Wainwright C (2006). Quality-of-life in children and adolescents with cystic fibrosis managed in both regional outreach and cystic fibrosis center settings in Queensland. J Pediatr.

[20] Coelho CC, Aquino Eda S, de Almeida DC, Oliveira GC, Pinto Rde C, Rezende IM, Passos C (2007). Comparative analysis and reproducibility of the modified shuttle walk test in normal children and in children with cystic fibrosis. J Bras Pneumol.

[21] Paranjape SM, Barnes LA, Carson KA, von Berg K, Loosen H, Mogayzel PJ (2012). Exercise improves lung function and habitual activity in children with cystic fibrosis. J Cyst Fibros.

[22] Hommerding PX, Donadio MV, Paim TF, Marostica PJ (2010). The Borg scale is accurate in children and adolescents older than 9 years with cystic fibrosis. Respir Care.

[23] Prasad SA, Randall SD, Balfour-Lynn IM (2000). Fifteen-count breathlessness score: an objective measure for children. Pediatr Pulmonol.

[24] Quittner AL, Modi AC, Wainwright C, Otto K, Kirihara J, Montgomery AB (2009). Determination of the minimal clinically important difference scores for the cystic fibrosis questionnaire-revised respiratory symptom scale in two populations of patients with cystic fibrosis and chronic Pseudomonas aeruginosa airway infection. Chest.

[25] Schmidt AM, Bregnballe V, Vebert Olesen H, Ingemann-Hansen T, Thastum M, Schiøtz PO (2010). Exercise and quality of life in patients with cystic fibrosis – a 12-week intervention study. J Cyst Fibros.

[26] Jones B, Jarvis P, Lewis JA, Ebbutt AF (1996). Trials to assess equivalence: the importance of rigorous methods. Bmj.

[27] Morris TP, White IR, Royston P (2014). Tuning multiple imputation by predictive mean matching and local residual draws. BMC Med Res Methodol.

[28] Sallis JF (1991). Self-report measures of Children's physical activity. J Sch Health.

