# Supplementary material for: CyFiT telehealth: protocol for a randomised controlled trial of an online outpatient physiotherapy service for children with cystic fibrosis
Source: BMC Pulm Med. 2019 Jan 24;19:21. doi: 10.1186/s12890-019-0784-z (PMC6344991; doi:10.1186/s12890-019-0784-z)
Supplement: Supplementary file 1 — Figure 1. Illustrates the current model of physiotherapy care for children with cystic fibrosis at our clinic. Prevention point is where the study intervention will be integrated into existing practices. (DOCX 133 kb) [file 12890_2019_784_MOESM1_ESM.docx]

#
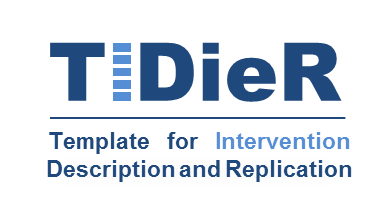
1.1 The TIDieR (Template for Intervention Description and Replication) Checklist*:

Information to include when describing an intervention and the location of the information

| **Item number** | **Item** | **Where located **** | |
| --- | --- | --- | --- |
|  |  | Primary paper  (page or appendix  number) | Other ^†^ (details) |
|  | **BRIEF NAME** |  |  |
| **1.** | CyFiT Telerehabilitation: A technology based physiotherapy to encourage peer driven participation in therapy, and quality of life. | Section 1.1 |  |
|  | **WHY** |  |  |
| **2.** | Managing Cystic Fibrosis is complex and disruptive to quality of life – especially for children. Telehealth offers a means of engaging children, and reduce burden associated with managing such complex disease. | Section 2.6 |  |
|  | **WHAT** |  |  |
| **3.** | Children managed using CyFiT Telerehabilitation, an online technology-based physiotherapy system. This can be accessed anyway with any devices with valid internet connection. CyFiT Telerehabilitation also uses the Garmin Vivosmart3® activity tracker to provide continuous, real-time information to clinicians. Through this platform, treating clinicians can deliver individual and group physiotherapy sessions, without risk of cross infection or the barrier of geographical distance. | Section 3a.11 |  |
| **4.** | 1. All: Tests at Start, 6 months, 12 months and 18 months: The 10 metre Shuttle Test (MST), and several questionnaires. 2. **CyFiT:** If your child has been allocated the CyFiT Group, your child will:  - Be given a Garmin vivosmart 3® activity tracker to wear every day, including sleeping (they are water and shower proof) and login information for the CyFiT website during your first session. You’ll both be shown how to use the device and the website. - You will continue any physiotherapy follow up and exercises as guided by your treating physiotherapist, any scheduled physio consultations will be conducted through the CyFiT website - you will not need to visit the hospital. - A supervised exercise class by your physiotherapist will be available on referral from your physiotherapist. - CyFiT will continue for 12 months, then you will return to usual physio care.  1. **Standard Group:** If your child has been allocated to the standard group, your child will:  - Continue their normal physiotherapy routine for the duration of the study i.e. current standard practice. - You will be asked to record dates of hospital physio visits or other physio reviews. | Section 3a.11 | Appendix 2.1  Appendix 3.1  Appendix 3.2 |
|  | **WHO PROVIDED** |  |  |
| **5.** | Treating clinicians will be cardiorespiratory physiotherapists from the at Lady Cilento Children’s Hospital, Children’s Health Queensland – Hospital and Health Services.  Assessments will be conducted by the Principal Research Investigators who are qualified physiotherapists. | Section 3a.11  Section 3c.18 |  |
|  | **HOW** |  |  |
| **6.** | Children allocated to CyFiT Telerehabilitation group will be managed remotely via telehealth, unless during unforeseen deterioration or illness.  Children in Standard Group will be managed through face to face clinical consultation. | Section 3a.11 |  |
|  | **WHERE** |  |  |
| **7.** | CyFiT Telerehabilitation is designed for families to undertake physiotherapy within their homes and local community.  Children Standard Group will be managed at Lady Cilento Children’s Hospital. | Section 3a.11 | Appendix 2.1  Appendix 2.3 |
|  | **WHEN and HOW MUCH** |  |  |
| **8.** | Assessment at Start, 6 months, 12 months and 18 months: The 10 metre Shuttle Test (MST), and several questionnaires.  Regular physiotherapy consultation and follow up will be decided between families and treating clinician | Section 3a.11 | Appendix 2.1  Appendix 2.3 |
|  | **TAILORING** |  |  |
| **9.** | Content of intervention for managing children with cystic fibrosis is completely determined by the clinical reasoning of the treating clinician based on the child’s presentation at time of review. This is due to the complexity of cystic fibrosis, and management is most effective when individualise | Section 3a.11 |  |
|  | **MODIFICATIONS** |  |  |
| **10.^ǂ^** | N/A at present |  |  |
|  | **HOW WELL** |  |  |
| **11.** | Planned: Outcome measures used in this study includes parent and child self-reported adherence but also activity data provided from Garmin vivosmart 3®. | Section 3a.12 |  |
| **12.^ǂ^** | Actual: N/A at present |  |  |

# 2.1 Parent Information Sheet

**Title**: CyFiT Telerehabilitation: Efficacy of a telehealth physiotherapy intervention to improve quality of life and participation in community based school-aged children with cystic fibrosis: a randomised controlled trial.

**Lay Title:** CyFiT - Technology based physiotherapy for children with CF

**It is OK to say NO – you can stop being in this study at any time**

**Thank you** for taking the time to read this information sheet. It is 4 pages long.

**What is an Information Statement?**These pages tell you about the study, and help you decide whether or not you would like your child to take part. Please read this information sheet carefully. You are welcome to ask us questions about anything in it. You may wish to talk about the project with your family, friends or a health care worker.

**Participation in this research is entirely voluntary.**If you don’t want your child to take part in the study, you don’t have to. You can withdraw your child at any time without explanation. Withdrawal will not affect your child’s care in any way.

**BACKGROUND – Why are we doing this study?**

Children with cystic fibrosis (CF) need a lot of time for physiotherapy and exercise. Families have very busy lives, and using telehealth may make things a bit easier. However there has been no research to confirm if telehealth is as good as face to face management for children with CF. CyFiT Telerehabilitation (CyFiT) is an engagement focused service that aims to improve children's participation, engagement, and accessibility to physiotherapy without compromise in fitness. CyFiT uses of latest technology and gadgets to engage and encourage participation to physiotherapy exercises in both individual and group settings.

**STUDY AIM – What are we planning to do?**

CyFiT will compare results of usual CF clinic management with children using technology for some appointments. We will use various measures such as physical function and quality of life in children from 8 to 18 years. There are no blood tests or invasive procedures in the study.

**RESEARCH QUESTIONS – What do we think we will find?**

We do not know if CyFiT Telerehabilitation will have the same results as usual CF care. We will have a better understanding of physical activity, quality of life, engagement in therapy, and costs.

**PARTICIPANTS**

**Why is my child being asked to be in this research project?**

We are asking your child to take part because he/she has cystic fibrosis and is aged between 8 and 18.

**What are the alternatives to taking part in this project?**

You can continue with usual CF management at the Lady Cilento Children’s Hospital if you choose not to participate.

**MEASURES – What will my child be doing in the study?**

If you agree for your child to participate in this study, your child will be randomly allocated to one of the two groups: CyFiT or Standard group. Only the CyFiT group will use the CyFiT Telerehabilitation service

Below is an outline for the procedure of the study:

1. All: Tests at Start, 6 months, 12 months and 18 months: The 10 metre Shuttle Test (MST), and quality of life questionnaires. Perform a physical activity and cough related reflection every week that takes approximately 1 minute.
2. **CyFiT:** If your child has been allocated the CyFiT Group, your child will:

- Be given a Garmin vivosmart 3 activity tracker to wear every day, including sleeping (they are water and shower proof) and login information for the CyFiT website during your first session. You’ll both be shown how to use the device and the website.
- You will continue any physiotherapy follow up and exercises as guided by your treating physiotherapist, any scheduled physio consultations will be conducted through the CyFiT website - you will not need to visit the hospital.
- A supervised exercise class by your physiotherapist will be available on referral from your physiotherapist.
- CyFiT will continue for 12 months, then you will return to usual physio care.

1. **Standard Group:** If your child has been allocated to the standard group, your child will:

- Continue their normal physiotherapy routine for the duration of the study i.e. current standard practice.
- You will be asked to record dates of hospital physio visits or other physio reviews.

All measurements will be performed by one of the investigators, or a staff member from the CF Clinic.

**TIME COMMITMENT – When and how often will this happen?**

To minimise any inconvenience to you, measurements will be performed when your child is already visiting the CF clinic at the Hospital. Tests will be done at the start, 6 months and 12 months (study period) and 18 months (follow-up period).

**PRIVACY & CONFIDENTIALITY**

**Privacy** – Your child’s privacy will be maintained at all times while participating in the study. Measurements will be performed in a private consulting room or in defined part of the outdoor area for the shuttle (beep) test.

**Confidentiality of Information** – Your child’s
information will not be known to anyone except
the researchers. Your child will be assigned a number with which all data sheets and files will be labelled. This number will be kept in a separate file so that research results and personal details are not together. Electronic images and files will be kept on a computer, where user access will be password protected. The computer and hard copies of the files will be kept in a locked filing cabinet in the University of Queensland. Information collected as part of this study may be used by investigators for future studies for which prior ethical approval and your consent will be sought.

**BENEFITS – Will this help your child and/or other children with CF?**

- We cannot guarantee or promise that your child will receive any benefits from this research.
- Your child will have a 1 in 2 chance of using a brand new activity tracker from Garmin for 12 months with no cost to you. When the study is finished you will receive a report of your child’s assessment results along with a summary of the study.

Possible benefits to all participants may include a better understanding of the impact of using telehealth and activity monitoring in young people with CF and their families.

**RISKS – Could anything bad happen to my child?**

- There will be no risk to your child greater than that of normal exercising at home and usual physiotherapy assessments
- Some muscle fatigue may occur with exercise tests – these are the same as your child’s usual management
- CyFiT Telerehabilitation is run by physiotherapists experienced in CF, and your child will be closely supervised. In the unlikely occurrence of adverse events or injuries, they would be reported and managed in the usual Children’s Health Queensland – Hospital and Health Service manner.

**CONSENT – What am I giving permission for?**

**Opting in** - You can decide whether or not to give permission for your child to be in this study. Your decision will not affect your child’s future relationship with the Lady Cilento Children’s Hospital.

**Opting out** - If you decide for your child to participate, you are free to withdraw your child from the study at any time without saying why. The decision to withdraw you child from the study will not affect their routine medical treatment or their relationship with the people treating them.

Further information – You can ask for further information before deciding to take part. For example, you may like to talk to your family, or with your child’s therapist or doctor.

**MORE INFORMATION**

If you would like further information, or if you have any questions regarding the nature of the research, please feel free to contact the chief investigator:

Mr (Ray) Lei Lang – 0422 193 168, lei.lang@uqconnect.edu.au

**Investigators:**

(Ray) Lei Lang PhD Candidate, The University of Queensland

Dr Leanne Johnston Senior Lecturer in Physiotherapy, The University of Queensland, School of Health and Rehabilitation Sciences

Prof Trevor Russell Co-Director, Centre of Research Excellence in Telehealth, The University of Queensland

Dr Kellie Stockton Director of Physiotherapy, Children’s Health Queensland, Lady Cilento Children’s Hospital

Christine Wilson Physiotherapy Consultant (non-acute), Children’s Health Queensland, Lady Cilento Children’s Hospital

**ETHICAL CLEARANCE**

This study has been cleared by the human research ethics committee of Children’s Health Queensland Hospital and Health Service, which operate in accordance with the National Health and Medical Research Council’s guidelines.

**Ethical Conduct**

If you have any concerns about the study about which you would rather speak to an Ethics officer of the Hospital not involved in the study, you may contact the Coordinator of CHQ Ethics Committee:

Ms Amanda Smith on 07 3069 7002 or email [CHQEthics@health.qld.gov.au](mailto:CHQEthics@health.qld.gov.au)

Thank you for your interest:

| **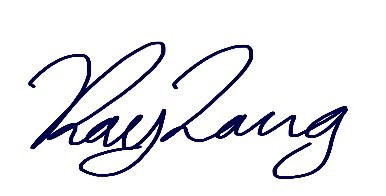** |
| --- |
| Mr (Ray) Lei Lang, Chief Investigator Date: 25 / 05 / 17 |

# 2.2 Parent/Guardian Consent Form

**Title:** CyFiT Telerehabilitation: Efficacy of a telehealth physiotherapy intervention to improve quality of life and participation in community based school-aged children with cystic fibrosis: a randomised controlled trial.

**Lay Title:** CyFiT - Technology based physiotherapy for children with CF

**Investigators:**

(Ray) Lei Lang PhD Candidate, The University of Queensland

Dr Leanne Johnston Senior Lecturer, The University of Queensland, School of Health and Rehabilitation Sciences

Prof Trevor Russell Co-Director, Centre of Research Excellence in Telehealth, The University of Queensland

Dr Kellie Stockton Director of Physiotherapy, Children’s Health Queensland, Lady Cilento Children’s Hospital

Christine Wilson Physiotherapy Consultant (non-acute), Children’s Health Queensland, Lady Cilento Children’s Hospital

**Parent/Guardian**

I, ________________________________________ (PLEASE PRINT) hereby
consent to

my child ____________________________________ (PLEASE PRINT) to take part in the above study.

I acknowledge that (a) I have read the information sheet provided and (b) the study, so far as it affects me/my child has been fully explained to my satisfaction by the investigators. After discussing the study with my child so that they understand and gaining their agreement to participate, I freely consent to my child’s participation in the project.

The details of the assessment and treatment procedures have been explained to us, including the anticipated length of time it will take, the frequency with which the appointments are to be carried out, and an indication of any discomfort or possible risks that may be expected.

(b) I / my child understand that the assessment
procedures to be undertaken in the project involve:

- Four, 1-hour assessment sessions linked to normal clinic times to measure my child’s physical fitness, and completing various questionnaires
- I will be asked about my views on my child’s therapy via short parent questionnaire
- My child may be allocated to have physiotherapy via telehealth for a total of 12 months.

(c) I/my child understand that my child may be allocated to the CyFiT Telerehabilitation Group to receive my current physiotherapy via CyFiT Telerehabilitation or the Standard Group to receive my current physiotherapy via standard protocol at Lady Cilento Children’s Hospital.

(d) I/my child understand that the purpose of this research is to gain information that will contribute to development of therapy services for children with cystic fibrosis and my child’s involvement may not be of any direct benefit to him/her.

(e) I/my child have been informed that the results of any tests involving him/her will not be published so as to reveal his/her identity and that his/her privacy will be maintained at all times.

(f) I/my child understand that I/my child is free to withdraw from this study at any stage without reason or penalty.

(g) I/my child understand that I may withdraw my child from the study at any time without giving reason for doing so and that their care at Lady Cilento Children’s Hospital will continue without prejudice.

(h) I/my child understand that this project follows the guidelines of the National Statement on Ethical Conduct in Research Involving Humans (2007) and that it has been approved by the ethics boards of Children’s Health Queensland Hospital and Health Service and The University of Queensland.

(i) I/my child understand that I can contact any of the investigators to discuss my child’s participation in the study at any time.

**I give consent for**(please tick as many as are appropriate):

**This study**

My child’s assessment data to be used for the current study

Images of the assessments performed in this study to be used in a de-identified way in research or educational presentations about this research

Signed: ______________________________ Date: ___ / ___ / ___
 (parent/guardian)

Telephone: ____________________________

Email: ________________________________

I would like to receive a copy of the study results

No  Yes, by email  Yes, by post

**Witness*** to parent / guardian signature:

Name of Witness to parent / guardian signature: ______________________________
 (PLEASE PRINT)

Signed: ______________________________ Date: ___ / ___ / ___

*The witness is not to be the investigator, a member of the study team or their delegate. If an interpreter is used, the interpreter may not act as witness to the consent process. Witness must be 18 years or older.

**Declaration by Senior Researcher**^β^

I have given a verbal explanation of the research project, its procedures and risks and I believe that the parent/guardian has understood that explanation.

Signed: ______________________________ Date: ___ / ___ / ___
 (Investigator)

^β^A senior member of the research team must provide the explanation of, and information concerning, the research project.

***Footnote*** ** Privacy guidelines of Queensland Health and the National Health and Medical Research Council (NHMRC) will be adhered to when data is added, stored or accessed.*

# 2.3 Child Information Sheet

**It is OK to say no – you can stop being in this study at any time**

**What is the study called?**CyFiT - Technology based physiotherapy for children with CF

**What is this information for?**This is about a research project. You can decide whether or not you want to be part of it. You can ask us questions about anything in it, and talk about the project with your Mum or Dad.

**It is your choice whether you do this research.**If you don’t want to take part, you don’t have to. You can stop being in the study at any time without telling us why. There will be no problem or change to your care in any way.

**What is the study about?**We are trying to find out new and fun ways to making of doing your physio exercises using everyday technology and the internet.

**What would you be doing in the study?**If you decide to take part in this study, when you come to the CF clinics, a researcher will:

- Measure your physical fitness (Shuttle test)
- Ask you and your Mum or Dad about the activities and exercises you do at home
- Answer some questions each week about your cough and activity

This will happen four times (straight away, after 6 months, after 12 months, and 6 months after the trial finishes) and will take about 1 hour, at your usual clinic visits.

Some people will do physiotherapy via the CyFiT website; others will continuing as usual with clinics and other follow-up. Some people will be allocated by a computer program to have a Garmin activity tracker and do web-based sessions. If you’re allocated to do web-based sessions, you won’t have to come into the hospital as often. This will continue to happen for 12 months (1 year).

**Could anything bad happen?**No, this is just like what you would normally at clinic. Your Mum and Dad can be with you the whole time (including the activities you do at home).

**Will it help me?**We can’t promise any special benefits of being in the study. You may get a better understanding of how you are managing CF yourself. We hope that the information from this study will improve the way we do physio for you in the future and for all the

other kids who have cystic fibrosis.

**Will I find out the results of the study?**You will hear your own results on each measurement day. You will be mailed a copy of the overall results of the study.

**Will anyone know about my results?**No. Only you and your parents and the researchers will know your results. We will never tell anyone else your name or show anyone else your results. If you and your parents agree, we might use some photos of you doing the activities when we are presenting the results of the research, but these photos will not include your face or your name, so they will not show who you are.

**Who is doing the project?**

From the Children’s Hospital: Physiotherapists from the CF clinic
Dr Kellie Stockton
Ms Christine Wilson

From the University of Queensland: Mr (Ray) Lei Lang
Dr Leanne Johnston
Prof Trevor Russell

**Would you like to know more?**You can ask for further information before deciding to take part. For example, you may like to talk with your family, or with your therapist or doctor.

Thank you for your interest in this research project! Please don’t forget to ask if you have any questions!

| **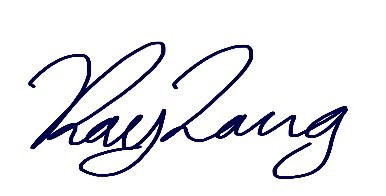** |
| --- |
| Mr (Ray) Lei Lang, Chief Investigator Date: 25 / 05 / 17 |

# 2.4 Child Assent Form

**What is the study called?**CyFiT Telerehabilitation - Technology based physiotherapy for children with cystic fibrosis

**Who is performing this project?**

Physiotherapists from the CF clinics, and Researchers from Children’s Hospital and The University of Queensland: (Ray) Lei Lang, Dr Leanne Johnston, Prof Trevor Russell, Dr Kellie Stockton, Ms Christine Wilson.

**Declaration by Child**

1. I, _____________________________________________

(PLEASE PRINT YOUR NAME)
agree to participate in the above research

| 1. I have read and talked about the information on this form. |  |
| --- | --- |
| 1. I know what will happen in the study and about how long it will take. |  |
| 1. I understand that this research is to help develop therapy services for children with CF, but it may not be of any direct benefit to me. |  |
| 1. I know that my information will be private and only used in the study without my name. |  |
| 1. I can stop being in this study at any stage without having to tell you why, and nothing bad will happen to me or my family. |  |

1. I give consent for (please tick as many as are appropriate):

This study

My results to be used for this study

Pictures of me to be used in research or educational
presentations – so long as they do not show who I am

Signed: ______________________________ Date: ___ / ___ / ___
 (child)

I would like to receive a copy of the study results

No  Yes, by email  Yes, by post

Signed: ______________________________ Date: ___ / ___ / ___
 (parent/guardian)

**Witness*** to child’s signature:

Name of Witness to child’s signature: _______________________________________
 (PLEASE PRINT)

Signed: ______________________________ Date: ___ / ___ / ___

*The witness is not to be the investigator, a member of the study team or their delegate. If an interpreter is used, the interpreter may not act as witness to the consent process. Witness must be 18 years or older.

**Declaration by Senior Researcher**^β^

I have given a verbal explanation of the research project, its procedures and risks and I believe that the parent/guardian has understood that explanation.

Signed: ______________________________ Date: ___ / ___ / ___
 (Investigator)

^β^A senior member of the research team must provide the explanation of, and information concerning, the research project.

***Footnote*** ** Privacy guidelines of Queensland Health and the National Health and Medical Research Council (NHMRC) will be adhered to when data is added, stored or accessed.*
